# User Engagement and Assessment of Treatment Effectiveness in Patients Using a Novel Digital mHealth App During Spinal Cord Stimulation Screening Trials

**DOI:** 10.2196/35134

**Published:** 2022-03-23

**Authors:** Jennifer M Lee, Rex Woon, Mandy Ramsum, Daniel S Halperin, Roshini Jain

**Affiliations:** 1 EvergreenHealth Pain Care Kirkland, WA United States; 2 Boston Scientific Neuromodulation Valencia, CA United States

**Keywords:** spinal cord stimulation, SCS, chronic pain, digital health, smartphone app, mobile health, mHealth, smart device, digital application, application, app, spine

## Abstract

**Background:**

Patient outcomes and experience during a Spinal Cord Stimulation (SCS) screening trial can have a significant effect on whether to proceed with long-term, permanent implantation of an SCS device for the treatment of chronic pain. Enhancing the ability to track and assess patients during this initial trial evaluation offers the potential for improved understanding regarding the suitability of permanent device implantation as well as identification of the SCS-based neurostimulative modalities and parameters that may provide substantial analgesia in a patient-specific manner.

**Objective:**

In this report, we aimed to describe a preliminary, real-world assessment of a new, real time tracking, smart, device-based digital app used by patients with chronic pain undergoing trial screening for SCS therapy.

**Methods:**

This is a real-world, retrospective evaluation of 13,331 patients diagnosed with chronic pain who used the new “mySCS” mobile app during an SCS screening trial. The app design is health insurance portability and accountability act (HIPAA)-compliant and compatible with most commercially available smartphones (eg, Apple, iPhone, and Android). The app enables tracking of user-inputted health-related responses (ie, pain relief, activity level, and sleep quality) in addition to personal trial goals and a summary of overall experience during the SCS trial. A deidentified, aggregate analysis of user engagement, user-submitted responses, and overall trial success was conducted.

**Results:**

When provided the opportunity, the percentage of users who engaged with the tracking app for ≥50% of the time during their trial was found to be 64.43% (n=8589). Among the 13,331 patients who used the app, 58.24% (n=7764) entered a trial goal. Most patients underwent SCS screening with a trial duration of at least 7 days (n=7739, 58.05%). Of those patients who undertook a 7-day SCS trial, 62.30% (n=3456) engaged the app for 4 days or more. In addition, among all who submitted descriptive responses using the app, health-related improvements were reported by 77.84% (n=10,377) of patients who reached day 3 of the screening phase assessment and by 83.04% (n=11,070) of those who reached trial completion. A trial success rate of 91% was determined for those who used the app (versus 85% success rate for nonusers).

**Conclusions:**

Data from this initial, real-world examination of a mobile, digital-health–based tracking app (“mySCS”), as used during the SCS screening phase, demonstrate that substantial patient engagement can be achieved while also providing for the acquisition of more real time patient-outcome measures that may help facilitate improved SCS trial success.

## Introduction

Widespread use of smart devices (ie, mobile phones and tablets) has fostered an unprecedented growth in the use of digital-based platforms and apps enabling real time tracking of health-related outcomes and experiences of patients undergoing treatment of chronic pain [1-3]. More importantly, these contemporary tools have been demonstrated to help instill a greater level of assurance in patients, that their pain condition can be controlled. These devices can also promote improved self-monitoring of the multidimensional experience of chronic pain, and some concurrently allow health care providers enhanced connectivity to real time outcomes reported by those under their care [4-6]. The implementation and use of new digital methodologies in the context of health care is underpinned by what is now referred to as ecological momentary assessment (EMA), an approach constituted by the frequent sampling of study subjects’ behavior, outcomes, and experiences in real time within the real-world environments in which they live and participate [7,8]. EMA, as a technique that relies on the repeated collection of data pertaining to the health-related condition of the patient, is therefore thought to reduce recall bias and enable improved assessment of the experience of patients with chronic pain. As such, patient-specific information, acquired in a spatiotemporal manner using EMA-based methods, may help provide better clinical assessment of individual patients given the highly subjective and variable experience of those having various chronic pain disorders.

Spinal Cord Stimulation (SCS) is an established treatment option for chronic pain, which delivers electrical impulses to neuronal tissues within or adjacent to the spinal cord. These impulses are capable of interrupting the transmission of dysregulated pain signals, typically due to nerve injury, which can occur between one or more localized anatomical areas (eg, low back, leg, foot, upper limb, etc) and the human brain. Typically, before patients are permanently implanted with an SCS device, they must first undergo a screening period, commonly termed as a trial, where they experience SCS therapy for a short duration of time (eg, up to 3-7 days) to assess whether the applied treatment is effective for reducing pain. Only on the basis of the experience and success of the trial, as determined by verbal reporting of significant pain relief (defined as a 50% or greater reduction in pain) as well as improvement in function, is a permanent device implanted for long-term use. However, SCS screening trials can be challenging given the difficulty that some patients have maintaining the engagement necessary to sufficiently assess whether their pain relief and functional goals are being effectively met or not due to complications or lack of successful outcomes, which are known to be associated with higher costs resulting from repeated attempts at management of chronic pain [9-12]. Thus, whether the ability to record and assess patient feedback in real time during this initial screening phase could allow for an improved experience for patients is an open question. As noted, various publications have previously reviewed and examined the use of mobile, digital health-based apps in patients with chronic pain [13]. However, to our knowledge, no published reports describing the use of a mobile, digital companion app during the SCS trial phase exist in the peer-reviewed literature. Here, we describe our initial, real-world evaluation of a simple, mobile, smart device-based app implemented as a tool to track user-submitted goals, health-related assessments, and satisfaction in those undergoing SCS screening in order to provide a more real time examination of the trial experience for patients with chronic pain. In so doing, we sought to also determine how capable the app is in eliciting patient engagement during this phase and whether this metric could be potentially correlative with SCS trial success.

## Methods

**Figure 1:**
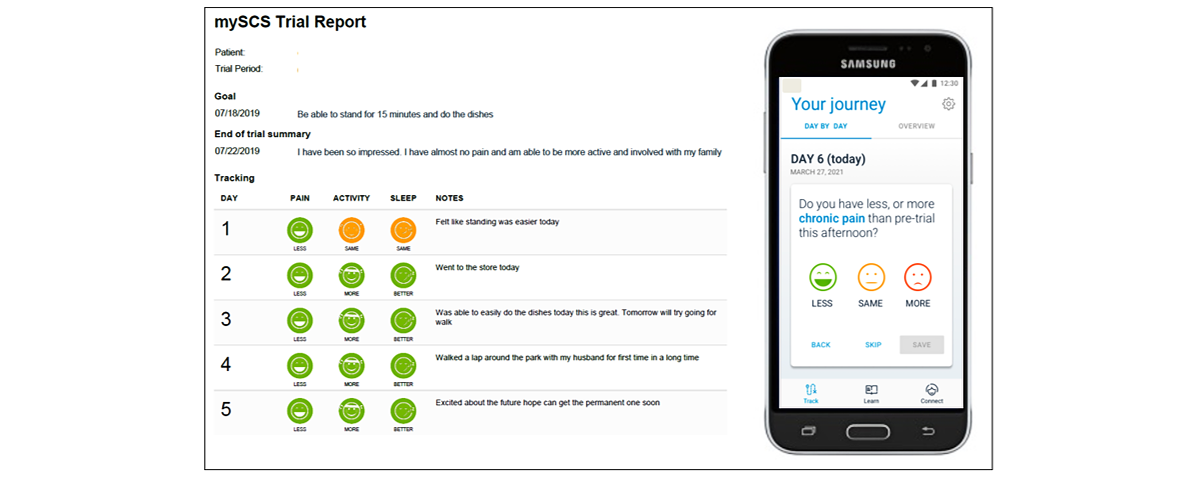
Pictorial representation of the mySCS app interface and sample trial report.

The newly commercially available mySCS app (Boston Scientific) was provided at no cost to patients diagnosed with chronic pain who participated in a trial (with a duration length up to 10 days) of an SCS system. The patients were invited to use the app either before or at their trial appointment. The patients were informed of the app either from a product brochure, their physician or physician office, or a company representative (Boston Scientific). The patients were presented with the opportunity to download the app during their SCS trial, but it was not a required condition in order to undergo their SCS trial. The patients were directed to carry out one of the following to help with the downloading process: search the app store to download, use a provided QR code to scan, or use an activation link. The QR code and activation links would take patients directly to the app listing on the app store to download the app. Company representatives were available at the trial appointment to assist with downloading if needed, but most patients were instructed to complete the installation before the day of their trial. The app was designed to be health insurance portability and accountability act (HIPAA)-compliant and was installed onto each participating patient’s personal smartphone (eg, Apple iPhone and Android) and is compatible with most recent smartphones and tablets. However, Android 8.0 or above and iOS 11 or later for iPhone, iPad, and iPod touch are required. The app is currently available on smart tablets, but not smart watches. All patients were required to provide consent to the terms of use following download and set up of the app. Figure 1 provides a pictorial representation of the app interface and a sample trial report that can be generated daily or at the end of the trial. In order to be eligible for inclusion, all patients were required to be least 18 years of age or older and have the following baseline demographic information available: trial start and end date, age, gender, and trial status (listed either as a “successful” or “failed” trial). Those patients listed with an “inconclusive” trial status and patients who underwent multiple trials were excluded from data analysis. Patients who were provided the app were instructed to use the app daily to record progress and their personal experience during the SCS trial. Patient-entered information was stored to a secure database that allows for exporting in PDF following completion of the trial. SCS screening trial data from a separate cohort of patients who did not use the app was also collected for comparative assessment. Gender and age demographic information was collected.

The mySCS app enables convenient tracking of information entered directly into the app by the patient. The patients were prompted to enter an assessment each day of their trial, which was of variable length, as determined by their physician. Typical trials range from 3 to 7 days, and the app reminds the patient to enter an assessment each day. Categorical descriptors selected in real time by patients were used to track ongoing user-submitted responses on a daily basis (vs patient’s pretrial condition) including intensity of chronic pain (“less,” “same,” and “more”), level of activity (“less,” “same,” and “more”), and sleep quality (“worse,” “same,” and “better”). Additionally, the patients entered their personal trial goal and overall trial satisfaction. User engagement with the digital app was defined as any user-submitted response, comment, goal, or assessment into the app. For each patient using the app, the number of days engaged with the app over the course of the length of the trial period was determined. Content analysis was carried out by assessing the frequency of terms entered into the app by patients. Recorded patient goals and summaries of trial satisfaction were assessed following completion of the SCS trial. Further, patient-entered trial satisfaction summaries were evaluated using a bigram analysis (occurrence of 2 consecutive words as a pair).

A successful SCS trial is conventionally defined as a ≥50% reduction in pain intensity at the end of the trial (vs pretrial pain intensity). Relative improvement in trial success between those who used the app (for at least 1 day) versus those who did not use the app was calculated by comparing the trial success rate between both of these separate cohorts using a one-tailed chi-squared test to determine if the proportions were different from each other at a statistical significance level of 0.05. All analyses were performed in Python (Python Software Foundation). The Pandas package was used for data management, and the Scipy package was used for performing statistical tests.

### Ethics Consideration

All data were collected in aggregate (independent of the centers in which patients were implanted) and was obtained fully deidentified, thereby obviating the need for ethics board review approval of this evaluation per United States 45 Code of Federal Regulations (CFR) § 46.104(d)(2)(i).

## Results

Data were acquired from a cohort of 13,331 patients who used the new mobile app during their SCS trial. Additionally, data from 12,196 patients who did not use the app were also obtained and evaluated. Gender and age demographics for those patients who did or did not use the app are shown in Table 1.

**Table 1:** Patient demographics (significant difference with chi-square test *P*<.001).

Characteristics	Patients with the mySCS app (n=13,331)	Patients without the mySCS app (n=12,196)
Gender—male, n (%)	5760 (43)	5648 (46)
Age (years), mean (SD)	60 (14)	66 (13)
Trial success, %	91	85

In total, 58.05% (n=7739) of those patients who had access and used the app underwent at least a 7-day trial with a maximum duration of up to 10 days. Initial engagement with the app required patients to enter in a personal goal for their SCS trial. Of the patients who used the app, nearly 58.24% (n=7764) were noted to have entered a trial goal. Analysis of the most prevalent key health-related functional terms occurring within the text of goals entered by patients using the mobile app is depicted in Table 2.

**Figure 2:**
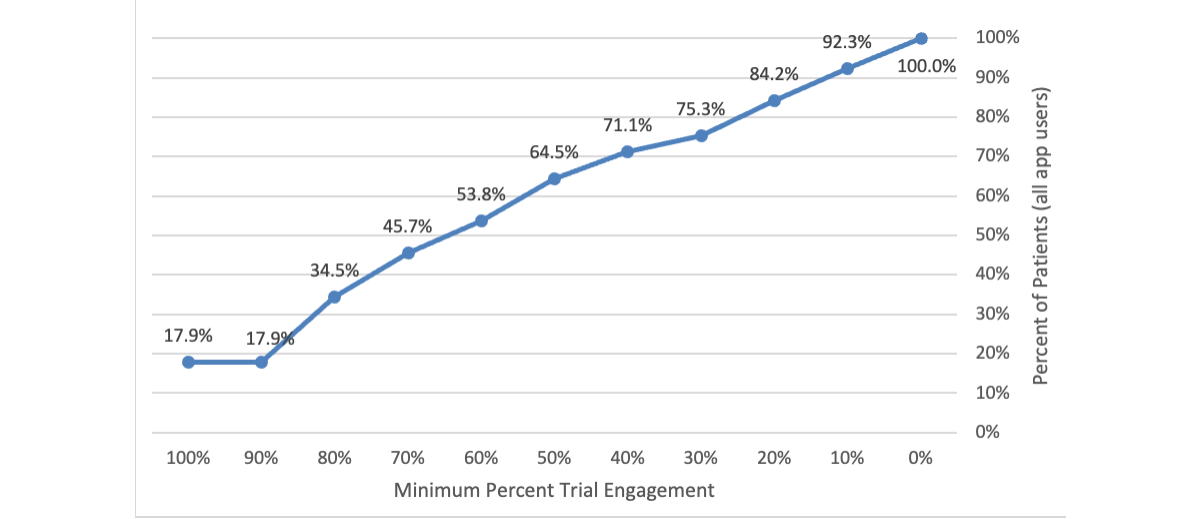
App user engagement during trial period.

The most common term (“walk”) was found in 46.81% (n=3634) of the entries provided by patients, followed by “less pain” (n=3514, 45.26%). Analysis of user engagement demonstrated that 64.43% (n=8589) of all users engaged the tracking app for at least 50% of the time within the total duration of their screening trial (Figure 2). Trials carried out for 7-days in duration were found to have been undertaken most frequently among those who used the app. Analysis of app engagement among those in this subcohort revealed that 62.3% (n=3456) of those who successfully completed a 7-day trial engaged the app for 4 days or more (Table 3).

Among all patients who used the app through day 3 and out to trial completion, 77.84% (n=10377) and 83.04% (n=11,070) demonstrated improvement in health-related metrics (ie, pain, activity level, and sleep quality), respectively. Of those patients who did not use the app, an 85% trial success rate (ie, ≥50% pain relief) was noted. Alternatively, a trial success rate of 91% was determined among those who did use the tracking app (Table 4) representing a 6% increase in trial success.

Evaluation of trial satisfaction summaries submitted by 1535 patients who used the app was conducted using a bigram analysis of the content that was recorded into the app following completion of the trial (Table 5). The most common consecutive 2-word phrase entered into the app by patients was found to be “less pain” followed by “more active” and “very well.”

**Table 2:** Occurrence of key terms in patient-entered goals (n=7795)

Key terms	Values, n (%)
Walk	3634 (46.81)
Less pain	3514 (45.26)
Sleep	1520 (19.58)
Stand	1265 (16.29)
Sit	730 (9)

**Table 3:** App engagement duration among users undergoing a 7-day trial (n=5547).

App engagement duration (days)	Patients with a 7-day trial, n (%)
≤1	954 (17.20)
2-3	1143 (20.61)
≥4	3450 (62.19)

**Table 4:** Trial success (≥50% pain relief) among app users and nonusers.

	Patients with successful trial, n (%)
Patients with the mySCS app (n=13,331)	12,133 (91.01)
Patients without the mySCS app (n=12,196)	10,359 (84.94)

**Table 5:** Occurrence of bi-grams in patient-entered trial assessments (n=1535).

Bi-gram	Trial assessments, n (%)
Less pain	108 (7.04)
More active	95 (6.19)
Very well	68 (4.43)
So much	62 (4.04)
Pain relief	59 (3.84)
Much better	57 (3.71)

## Discussion

### Principal Findings

This report, to our knowledge, is the first to demonstrate that successful patient engagement and capability for real time assessment of SCS trial outcomes can be achieved using a new, HIPAA-compliant, digital health-based mobile tracking app (“mySCS”). In addition, the use of this app was found to be associated with an increased proportion of SCS trial success. We also observed that patients using this new app on their smart device were able to better recognize their responses to treatment (compared to patient recall), while also enabling the progress of patients to be more clearly communicated after the trial period. This is a potentially important finding given the lack of reliability and the well-known difficulty that patients are known to have when using memory-based recall to estimate the intensity of the pain they experience [14-16].

In accordance with previous reports that have shown that patients with chronic pain are strongly interested and eager to use a digital health-based mobile app, we found that a large percentage of patients undergoing an SCS trial, if offered, are willing to use such a tool [4,6,8]. Given the importance the trial phase can have on the decision to proceed with permanent implantation of an SCS device, this sizable rate of user participation demonstrates the potential viability of a mobile tracking app to improve the utility of an SCS trial. This result is encouraging given that a substantial portion of the SCS-implanted population is over the age of 65 years and often beset by psychosocial factors that can affect patient function shown to be correlated with less successful trial outcomes [17,18]. Furthermore, in surveys conducted prior to the COVID-19 pandemic, those aged 65 years or older were found to have had substantially less knowledge or experience with digital health apps [19-21]. Interestingly, in accordance with more recent reports assessing user engagement with virtual health tools during the COVID-19 pandemic, 2 published studies by Han et al [22] and Lu et al [23] (both of which were conducted during the height of the recent COVID-19 pandemic) reported the successful implementation of remote monitoring and programming of those implanted with an SCS device; they also found that an overwhelming majority of patients had a strong desire for follow-up visits that do not require in-person interaction with their health care provider [22-25]. The results from these early investigations and of those now presented in this report thus jointly suggest that the use of digital health technologies that can track and record SCS outcomes and experience are likely to be highly desired and used by patients, including potentially even those >65 years of age, as a consequence, at least in part, of the necessities imposed on older adults to become more accustomed to remote-accessible tools during the COVID-19 pandemic. Moreover, it has also been postulated that costs associated with the implementation of SCS for treatment of chronic pain may be lowered when using digital health tools that allow for remote-based patient tracking and follow-up visits [22,23]. Though clinical examination (and publication of data) pertaining to the use of digital health-based tools in the context of SCS therapies is still quite limited at this time, these initial reports would suggest that the benefit to patients and providers (with the potential of integrating the use of new digital technologies as a part of an SCS-based therapeutic regimen for chronic pain) could be potentially substantial. We further surmise that the gradual shift toward more ubiquitous use of various digital tools in the real-world clinical setting may likely facilitate the eventual incorporation of digital health-based technology as a key component of the routine care provided to patients within the practical context of interventional pain management (to better monitor and treat those implanted with an SCS device in a more remote and personalized manner).

Patients using the new mobile app examined in this evaluation demonstrated an improved rate of trial success (vs a separate cohort of those who did not use the app). However, it is currently unknown if patient satisfaction at completion of an SCS trial is in fact greatly altered in patients implanted with an SCS device for chronic pain who use a digital health-based tool versus those who do not. Nonetheless, an extrapolation of the data described in Table 4 of this report indicates that, had those who did not use the app during their SCS trial chosen to do so, up to an additional 732 patients (calculated per the difference between 91% of app users and 85% of non–app users who had trial success) could have potentially achieved a successful trial. Interestingly, other reports of patients using implantable systems, such as Deep Brain Stimulation devices, have shown that a measurable improvement in outcomes (eg, patient satisfaction) can be observed in those undergoing remote monitoring using digital health technologies [26-28]. These notable improvements are thought to be due, at least in part, to an increase in the positive impression of treatment and overall psychological benefit that patients obtain when they log their progress routinely and reflect on their current health-related state. Mental health and treatment expectations are thought to have at least some effect on outcomes in most patients treated with SCS for chronic pain [18,29,30]. Therefore, whether the use of available digital health tools equipped with EMA-based tracking or remote-based communication features is, in turn, correlated with improved clinical outcomes (eg, psychological health measures) is now an important question warranting further investigation.

### Limitations

Given the preliminary nature of the evaluation described, limitations associated with the analyses described in this report must be noted. First, assessment of data was conducted retrospectively on the basis of the initial real-world launch of the tracking app made available to SCS-implanted patients. Future investigations are now needed to prospectively examine the impact of new digital health tools on patient outcomes using predefined measures and study designs including those that address the role that treatment expectations (ie, placebo responses) may have on obtained clinical outcomes. Additionally, the version of the app used by those described in this report did not allow for a highly detailed recording of baseline demographics, procedural information, or pain intensity based on an established rating scale (eg, Visual Analogue Scale). Going forward, procurement of such patient information may facilitate the detection of any selection bias (ie, bias as a result of the inadvertent selection for a particular patient segment within the overall cohort of assessed individuals such that the sample evaluated is not truly representative of the actual intended patient population) among those who used the app versus those who did not use the app. Information as it pertains to medical history, lead location, spinal level placement, and applied stimulation parameters of those who used the app may also have provided further insight as to the presence of any correlations associated with patient engagement and trial success. Moreover, no data were available that would have allowed an analysis of the percentage of patients who continued to receive a permanent implant after their successful trial or whether specific goals beyond pain relief alone (eg, improvements in functional disability) were achieved. Rates of conversion from trial to permanent implant are thought to have implications regarding overall device efficacy as well as other aspects related to the successful use of SCS as a therapeutic option for chronic pain [31]. As such, examination of this key metric in patients using digital health tools as part of an SCS trial, such as a future version of the mobile tracking app described in this report, is now warranted.

### Conclusion

This initial, real-world examination of a real time, mobile, digital-health–based tracking app (“mySCS”), as used during the SCS trial, demonstrates that substantial patient engagement can be achieved while also providing for more reliable and quantitative outcome measures that may help facilitate increased SCS trial success. The use of a novel digital-health–based mobile app therefore may constitute an important new approach toward fostering an improved experience during the SCS trial. A greater understanding of patient-specific clinical responses may also allow for better decision-making and evaluation regarding the appropriate use and effectiveness of SCS as a therapeutic strategy for treatment of chronic pain. Additional study and assessment are now needed to further understand the potential benefits of digital-health–based tools in the context of SCS therapy.

## Abbreviations

EMA: ecological momentary assessment
HIPAA: health insurance portability and accountability act
SCS: spinal cord stimulation
